# *Echinococcus multilocularis* inoculation induces NK cell functional decrease through high expression of NKG2A in C57BL/6 mice

**DOI:** 10.1186/s12879-019-4417-1

**Published:** 2019-09-09

**Authors:** Abuduaini Abulizi, Yingmei Shao, Tuerganaili Aji, Zhide Li, Chuanshan Zhang, Abudusalamu Aini, Hui Wang, Tuerhongjiang Tuxun, Liang Li, Ning Zhang, Renyong Lin, Hao Wen

**Affiliations:** 1 State Key Laboratory of Pathogenesis, Prevention and Treatment of High Incidence Diseases in Central Asia, Hepatobiliary & Hydatid Disease Department, Digestive & Vascular Surgery Center, First Affiliated Hospital of Xinjiang Medical University, Urumqi, 830054 China; 2 WHO Collaborating Center on Prevention and Management of Echinococcosis, First Affiliated Hospital of Xinjiang Medical University, Urumqi, 830054 China; 3 Xinjiang Key Laboratory of Fundamental Research on Echinococcosis, Clinical Medical Institute, First Affiliated Hospital of Xinjiang Medical University, Urumqi, 830054 China; 4 Department of Liver and Laparoscopic Surgery, Digestive & Vascular Surgery Center, First Affiliated Hospital of Xinjiang Medical University, Urumqi, 830054 China

**Keywords:** *Echinococcus multilocularis*, NK cells, NKG2A, Immune tolerance

## Abstract

**Background:**

Alveolar echinococcosis (AE) is caused by the larval stage of *Echinococcus multilocularis* (*E. multilocularis*), and considered as public health issue. Parasite-host immune interaction is pivotal during infection. As a subset of innate lymphoid cells, NK cells are known to play an important role during virus, bacteria, intra/extracellular parasitic infections and tumor progression. However, the possible role of NK cells in *E. multilocularis* infection in both human and murine is little known. Herein, the functional alteration of hepatic NK cells and their related molecules in *E. multilocularis* infected mice were studied.

**Methods:**

2000 protoscoleces (PSCs) were injected to C57BL/6 mice via the portal vein to establish secondary *E. multilocularis* infection*.* NK cells population and their related molecules (CD69, Ly49D, Ly49G2, Ly49H, Ly49I, NKG2A, NKG2D, granzyme B, IFN-γ, TNF-α) were assessed by using fluorescence-activated cell sorter (FACS) techniques and qRT-PCR. NK cell depletion was performed for further understanding the possible function of NK cells during infection.

**Results:**

The total frequencies of NK cells and NK-derived IFN-γ production were significantly reduced at designated time points (2, 4, 12 weeks). The liver resident (CD49a^+^DX5^−^) NK cells are decreased at 4 weeks after inoculation and which is significantly lower than in control mice. Moreover, in vivo antibody-mediated NK cell depletion increased parasitic load and decreased peri-parasitic fibrosis. Expression of the inhibitory receptor NKG2A was negatively related to NK- derived IFN-γ secretion.

**Conclusions:**

Our study showed down regulates of NK cells and upper regulates of NKG2A expression on NK cells during *E. multilocularis* infection. Reduction of NK cell frequencies and increased NKG2A might result in low cytotoxic activity through decreased IFN-γ secretion in *E. multilocularis* infection. This result might be helpful to restore NK cell related immunity against *E. multilocularis* infection to treat alveolar echinococcosis*.*

**Electronic supplementary material:**

The online version of this article (10.1186/s12879-019-4417-1) contains supplementary material, which is available to authorized users.

## Background

Alveolar echinococcosis, caused by the larval stage of *E. multilocularis*, continues to be a real world-wide public health issue. It is prevalent mainly in Western China, Middle East and as well as Central Europe [1]. China harbors nearly 90% of economic burden of AE around the world and thus sustained efforts have been made on prevention, control and management of this disease [2]. *E. multilocularis* infection predominantly target itself in the host’s liver and reside itself with incoming infiltrative growth and consequently lead to the critical involvement of vasculature [3]. Although, tremendous improvement has been made in the field of hepatic surgery including radical resection, liver transplantation and ex vivo liver resection and autotransplantation with promising clinical outcome [3]. Of note, nearly 90% mortality rate was reported within 10~15 years after initial diagnosis if untreated or insufficiently treated [4–6]. The attempt to unveil the underlined mechanism of such an infiltrative disease, regarded as “parasitic cancer”, is vital important.

To date, AE is considered as immune related parasitic infection with very intriguing and diversified immune cross-talk between host and parasite depending on the stage of the disease [7]. It is reported the *E. multilocularis* infection modulate Th cell subsets to maintain a high Th1 in early stage while Th2 dominant immune profile in both peripheral and regional milieu [8]. Our recent studies have demonstrated the potential importance of the remaining Th subsets such as Th17 [9], Treg [10] and Th9 [11] in *E. multilocularis* infection. Besides, our data indicated T-cell tolerance and exhaustion during clearance of *E. multilocularis* [12]. CD4^+^T and CD8^+^T cells present the major source of T cells in early and late stage of *E. multilocularis* infection, respectively [13]. Other studies indicate that the early infective stage of *E. multilocularis* is a strong inducer of tolerance in dendritic cells (DCs) [14], and the proliferative potential of the parasite metacestode tissue is dependent on the peri-parasitic immune-mediated processes of the host [7].

The both adaptive and innated immunity is pivotal importance to the parasite infection [15]. As an active member of innate immunity, NK cells compose approximately 20–30% of liver-resident lymphocytes with the far lower percentage in peripheral blood [16]. The contact-dependent signals provided by DCs, monocyte/macrophages, CD4^+^T cells as well as secreted cytokines activate NK cells during various infections [17]. It causes death of virus-infected cells [18, 19], tumor cells [20], and limit the progression of intracellular and extracellular parasites [21–25]. It is also reported that, the liver fibrosis and carcinogenesis formation process is hugely limited in the presence of NK cells in hepatitis [26]. Preliminary data showed the inhibited activation and proliferation of NK cells in *E. multilocularis* vesicular fluid co-culture and indicated its possible role in tolerative pathogen-host interaction [27].

Although, a plenty of work has been done in the field of immune interaction in *E. multilocularis* infection, however, very few is known regarding the possible role of innate immunity, especially NK cells in *E. multilocularis* infection. Herein, we are aiming to explore the expression of NK cells and its relative molecules, its potential impact on the disease progression, if any, in murine model of portal vein inoculation of *E. multilocularis.*

## Methods

### Animal model and infection

Eight-to-ten weeks old C57BL/6 female mice (Beijing Vital River Experimental Animal Technology Co. Ltd.), raised with standard feed and water in sterilized fifty-fifty light/dark altered condition, were used as animal model for *E. multilocularis* protoscoleces (PSCs) infection. PSCs, which was intraperitoneally carried within lesions in BALB/c mice prior to acquisition, was cleaned-up for several times by phosphate buffered saline (PBS, pH = 7.2, containing 1000 mg/mL penicillin and 1000 U/mL streptomycin) to prepare an injectable and sterilized suspension. The number of PSCs in the suspension was counted (using a DMI 4000B microscope, Leica, Germany), and) and adjusted by sampling three times to proper PSCs concentration in EP tubes before injection. Mice were anesthetized by 2.5% chloral hydrate and each mouse injected 0.1~0.15 ml into the abdominal cavity. Mice were inoculated via the hepatic portal vein with 2000 doses of PSCs in saline, whereas control mice were injected with isotonic saline. All mice received 200–300 μl of *E. multilocularis* PSCs sediment via the hepatic portal vein by using a 0.45 × 15RWLB venous infusion needle [12]. Mice were sacrificed at every experimental timepoints (2, 4, 12, 24 weeks after model was established) using euthanasia, which was approved by Institutional Animal Care and Use Committee. Specifically, mice were intraperitoneally (left lower quadrant) injected with 5% chloral hydrate 0.15–0.20 ml/mouse through medical syringe needle, and peripheral blood was collected by retro-orbital bleeding after successfully anesthetized; then, whole liver samples were obtained surgically; at last, mice were euthanized using cervical dislocation; the dead body further packed with special medical waste bags to freeze under − 20 °C and delivered for biosafety handling. Cysts were established at least 4 weeks after *multilocularis* inoculation, infections were confirmed based on both gross specimen observations and microscopic evaluation when the mice were sacrificed, and parasitic load in the livers were determined using cyst or lesion scales measured when liver specimens were obtained. That week number 2–4, 4–12 and 12–24 were respectively divided as pre-, intra-, and post-encystment phases in our team’s previous publication [12] that used same methodology. We designated these phases: 2 weeks after inoculation as pre-encystment timepoint (early stage); 4 and 12 weeks after inoculation as intra-encystment timepoints (middle stage); 24 weeks after inoculation as post-encystment timepoint (late stage).

### Flowcytometry analysis

Hepatic monocular lymphocytes were isolated as described [28]. For flowcytometry, after incubation with Fc-receptor blockage for 15 min at 4 °C, NK1.1, CD3, CD69, CD27, CD11b, CD49a, DX5, Ly49D, Ly49G2, Ly49H, Ly49I, NKG2A, NKG2D, granzyme B, IFN-γ, TNF-α molecules were marked with relevant antibodies according manufactory protocols (Additional file 1: Table S1) in order to analysis of some inhibitory/activating molecules and cytokines dynamics on NK cells. Moreover, as for intracellular cytokine marking, approximately 1 × 10^6 monocular lymphocytes were stimulated using Cell Stimulation Cocktail for 4 h [28]. IgG isotype controls were used as parallels. At last, we applied ALSRFortessa flow cytometry platform and Flowjo Software to analyze output data.

### Quantitative real-time PCR (qRT-PCR) analysis

Quantitative RT-PCR was used for analysis the mRNA levels of CD69, NKG2A, NKG2D, Ly49G2, Ly49I, Ly49D, Ly49H in whole-liver tissue at designated time-points after infection with *E. multilocularis* and normalized by comparison to GAPDH.

Total RNA, extracted from the liver using Trizol Reagent (Invitrogen, Canada), was reversed using M-MLV transcriptase according to manufactures’ instructions (Thermo, USA), then qRT-PCR (see primer information in Table 1) was implemented in thermocycler (iQ5 Bio-Rad, Canada) with SYBR Green PCR premix (TaKaRa, China) [29]. The results were calculated by the 2^−△△Ct^ method.

**Table 1:** Sequences of the qRT-PCR primers

Gene	Forward primer	Reverse primer
CD69	TGGTCCTCATCACGTCCTTAATAA	TCCAACTTCTCGTACAAGCCTG
NKG2A	TTCAGCACAGCCTTGTCCTC	CTTCTTTCCAGACCCAGGGC
NKG2D	CCAATGTTCGTTGTTCGAGTCC	GCACAATACTGGCTGAAACGTC
Ly49G2	TGCCACGATAACTGCAGCC	ATGGGTCTTTTGTGAACACCTG
Ly49I	GGAACAGTGAAACCAAGACGG	CTGTAATGCTGGCAGTTCGC
Ly49D	TTCAGGGTTGCAGAACGAGATGAG	AGGATCCCGAGAGCTATCACAATG
Ly49H	TGGGACAGTGAAACCAAGAGTG	GCTGTAATGCTGGCAGTTCG
GAPDH	CACTCACGGCAAATTCAACGGCAC	GACTCCACGACATACTCAGCAC

### NK cell depletion in vivo

NK cells were depleted by administering 150 μg anti-NK1.1 (clone PK136, BioXCell) or control (PBS) via intraperitoneal injection three times a week, which started on 12th week after *E. multilocularis* PSCs inoculation. The efficacy of NK cells depletion was verified by flowcytometry of PBMC after depletion.

### Routine histopathological observation and collagen staining

Peri-foci histopathological changes were evaluated in three different levels: (a) inflammatory foci: accumulation of inflammatory cells to form small foci, but free of parasite, visible PSCs or metacestodes; (b) inflammatory foci with fibrosis: fibrotic tissue formation on the basis of (a); (c) infectious foci with germinal layer: accumulation of inflammatory cells and fibrosis with obvious germinal layer of the parasite [12]. Infection status was assessed by the percentage of above three levels’ quantity within the liver. Peri-foci fibrosis determined by picric acid-sirius red staining and immunohistochemical staining for α-SMA for detection of activated hepatic stellate cells (HSCs) (original magnification× 100). The percentage of positive staining areas and cells was calculated using cellSens Dimension software (Olympus, Tokyo, Japan).

### Statistical analysis

Statistical analysis was performed using GraphPad Prism 7.0 (GraphPad Software, San Diego, CA). Tow-way ANOVA test with a Tukey’s multiple comparison was used when there were more than two groups. Sample size: 3–5 mice per group. *P* < 0.05 was considered statistically significant. (*P* -values were presented as: * *P* < 0.05; ** *P* < 0.01; *** *P* < 0.001; **** *P* < 0.0001).

## Results

### NK cells population is markedly decreased in infected mice

**Fig. 1:**
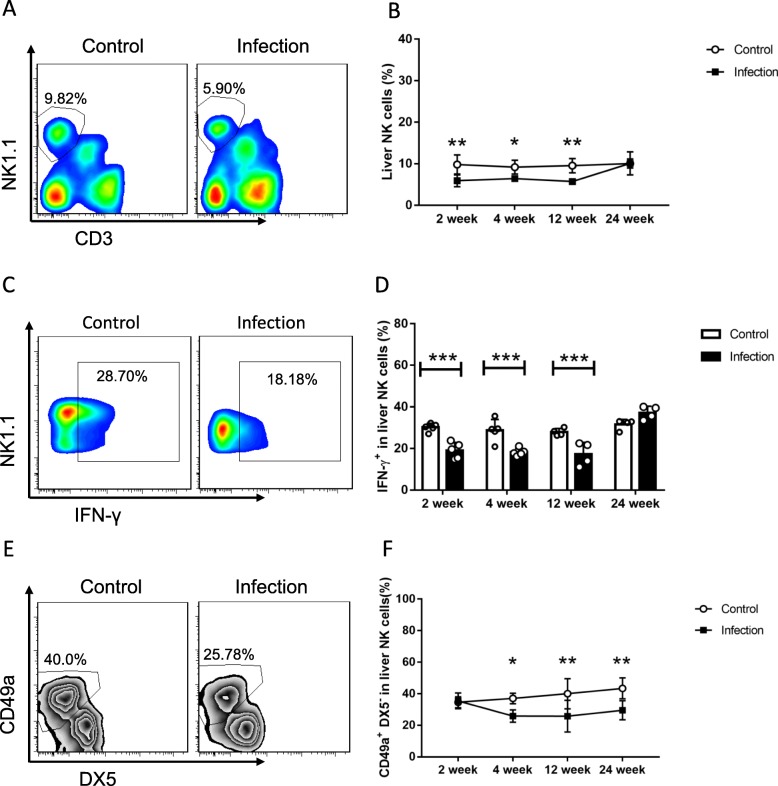
The decline in liver NK cells percentage and function in *E. multilocularis* infection. **a** Representative FACS plots gated on percentage of liver NK cells after infection. **b** Percentage of liver NK cells during the different time-points after infection. **c** Representative FACS plots gated on percentages of liver NK cells secretion of IFN-γafter infection. **d** The percentages of liver NK cells secretion of IFN-γ during different time points after infection. **e** Representative FACS plots gated on percentage of CD49a^+^DX5^−^ NK cells in liver NK cells after infection. **f** The percentage of CD49a^+^DX5^−^ liver resident NK cells during the different time points after infection. Data were shown as mean ± standard error (SEM, 4–6 mice per group), *p < 0.05, **p < 0.01, ***p < 0.001

**Fig. 2:**
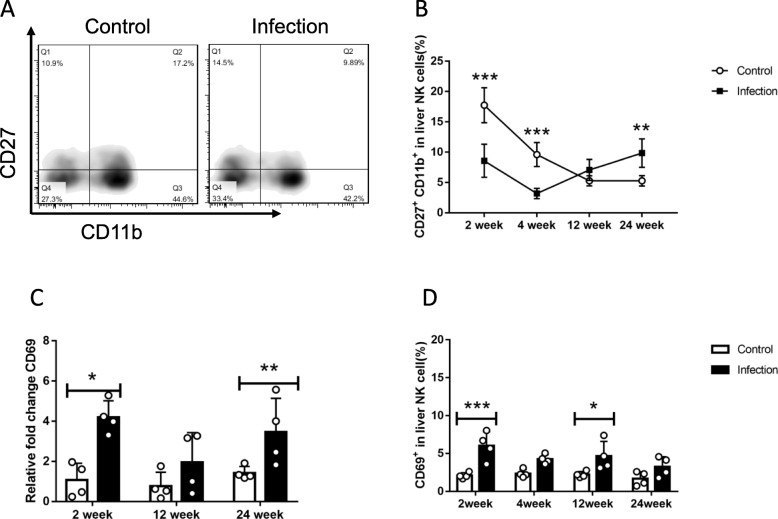
The maturation status and activation of mice liver NK cells in *E. multilocularis* infection. **a** Representative FACS plots gated on percentage of CD27^+^CD11b^+^NK cells in liver NK cells at 2 weeks after infection. **b** Percentage of mature CD27^+^CD11b^+^ in liver NK cells at different time points after infection. **c** QRT-PCR analysis for mRNA levels of receptor CD69 in whole-liver tissue at various time points after infection and normalized by comparison to GAPDH mRNA. **d** Percentage of CD69^+^ NK cells in liver at different time points after infection. Data were shown as mean ± standard error (SEM, 4–5 mice per group), *p < 0.05; **p < 0.01; ***p < 0.001; ****p < 0.0001

The hepatic NK cells population and IFN-γproduction were decreased respectively at 2, 4, 12 weeks post *E. multilocularis* inoculation, but at 24th week after infection no change was observed (Fig. 1a, b, c, d), while TNF-α and granzyme B levels had no significance in designated time points (Additional file 2: Figure S1). The percentage of liver resident CD49a^+^DX5^−^ NK cells gradually decreased at 4 weeks after *E. multilocularis* infection (Fig. 1e, f). It is currently known, NK cells move through four distinct maturation steps marked by changes in expression of CD27 and CD11b [30]. Given that background, we observed CD27 and CD11b changes in order to determine whether the reduction of NK cells population was due to maturation dynamics or activation process. Our results showed that, NK cells transformed from an immature form (CD27^−^CD11b^−^, stage 1) into mature or older forms (CD27^−^CD11b^+^, stage 4) during *E. multilocularis* infection (Fig. 2a). Further, the most functional subtype CD27^+^CD11b^+^ (stage 3) comparatively decreased at early stages (2, 4 weeks), while increased at middle and late stages (12, 24 weeks) after infection (Fig. 2b). Besides, during the whole process, CD69-positive NK cells ratio was elevated compared to control mice (Fig. 2c, d). Theses above results illustrated that *E. multilocularis* infection can modulate both hepatic NK cells population and its’ IFN-γproduction processes.

### NK cell depletion in vivo exacerbated *E. multilocularis* infection

**Fig. 3:**
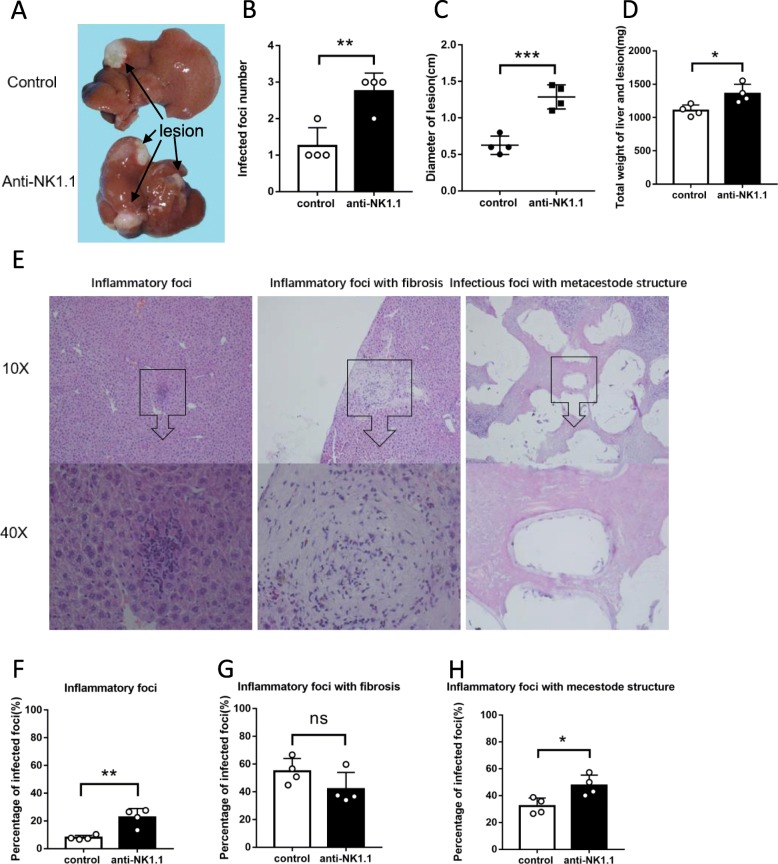
Hepatic histopathological alterations and granulomatous response in *E. multilocularis* infection while in vivo depletion of NK cells population. **a** The macroscopic views of the hepatic lesions infected mice. **b** The number of intrahepatic *E. multilocularis* infected lesions. **c** The sum of diameters for lesions in liver. **d** Total weight of the liver and infected hepatic lesions. **e** Histopathological alterations in liver of infected mice. H&E staining of liver sections. The original magnification was at 10×, and the below corresponding images were magnified at 40×, respectively. **f**, **g** and **h** Hepatic granulomatous response to *E. multilocularis* infection [12]. Data were shown as mean ± standard error (SEM, 4 mice per group), *p < 0.05. **p < 0.01, ***p < 0.001

**Fig. 4:**
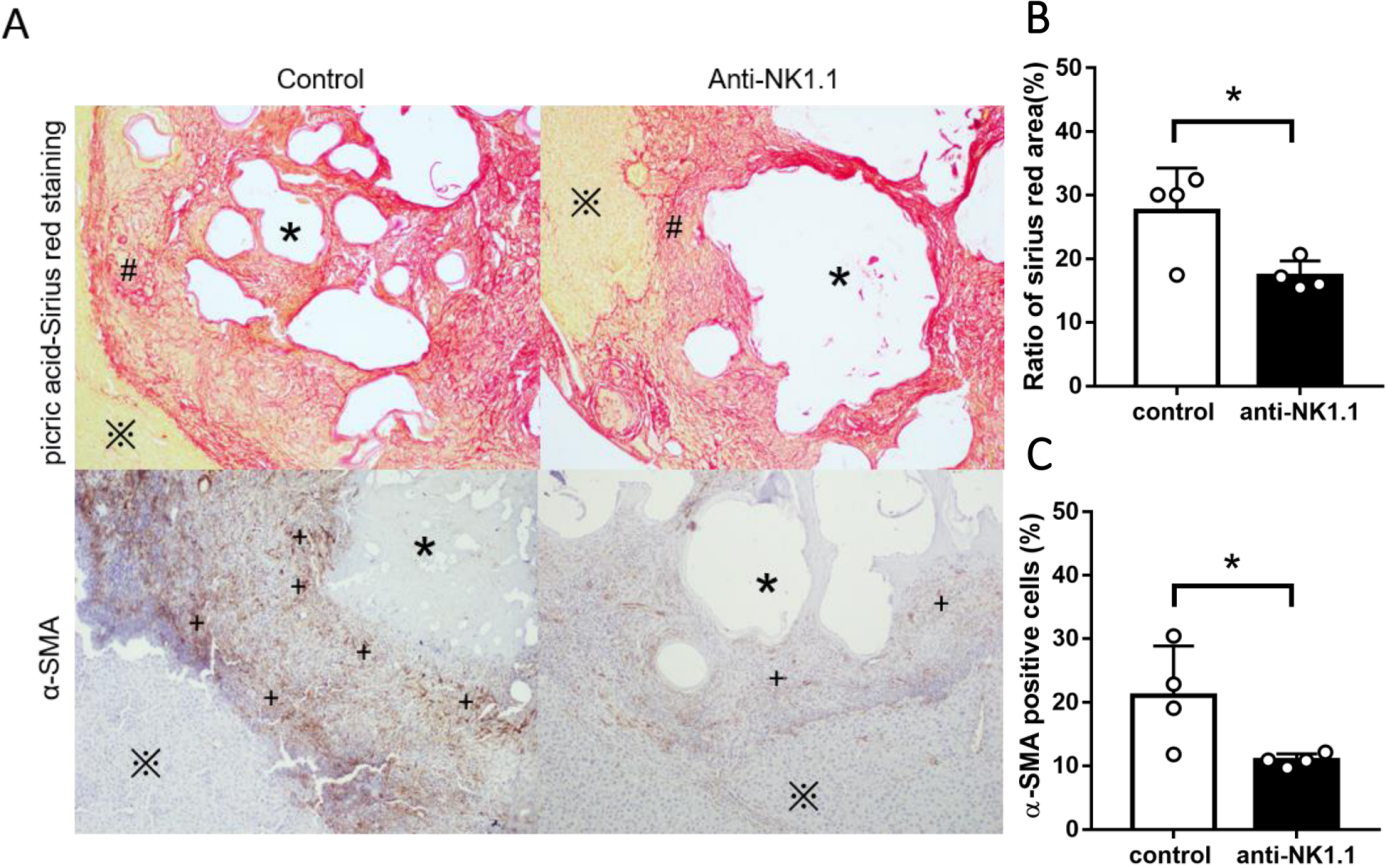
The peri-parasitic fibrosis in *E. multilocularis* infected mice while in vivo depletion of NK cells population. **a** The peri-parasitic fibrosis of hepatic lesion was determined by picric acid-Sirius red staining (the red area represents fibrillar collagen) and immunohistochemical staining for α-SMA (detection of activated hepatic stellate cells) (original magnification× 100). **b** The peri-parasitic fibrosis area of the section was quantified using cellSens Dimension software, the ratio of collagen area and total area (%) was counted. **c** The percentage of positive staining cells in peri-parasitic area was calculated using cellSens Dimension software to assess the expression of α-SMA on the lesion around areas. Lesion (*), liver tissue (**※**), peri-parasitic fibrosis area (**#**), activated hepatic stellate cells (**+**). (Data were shown as mean ± standard error (SEM, 4 mice per group), *p < 0.05

To clarify NK cells’ overall functional role, in vivo antibody mediated NK cell depletion was performed. The intrahepatic parasitic load was increased when NK cell was depleted (Fig. 3a, b, c, d). Specifically, the percentage of inflammatory foci and infectious foci with metacestode structures were higher than control mice (Fig. 3e, f, h). Peri-lesion (infected foci) collagen deposition, assessed by Sirius Red Staining and immunohistochemical staining for α-SMA for detection of activated hepatic stellate cells, revealed obvious reduction of peri-parasitic fibrosis and increase of intrahepatic parasitic load in NK cell-depleted mice (Fig. 4). These results suggested that, NK cell depletion might have downregulated cytotoxic activity of immune cells in peri-lesion microenvironment and fibrotic structure formation, which together helped with the pathogen to easily progress in the liver and to upgrade lesion load. However, it’s still unclear that what molecule or mechanism led theses changes in NK cells.

### Alteration of both activating and inhibitory NK cell receptors in infected mice

**Fig. 5:**
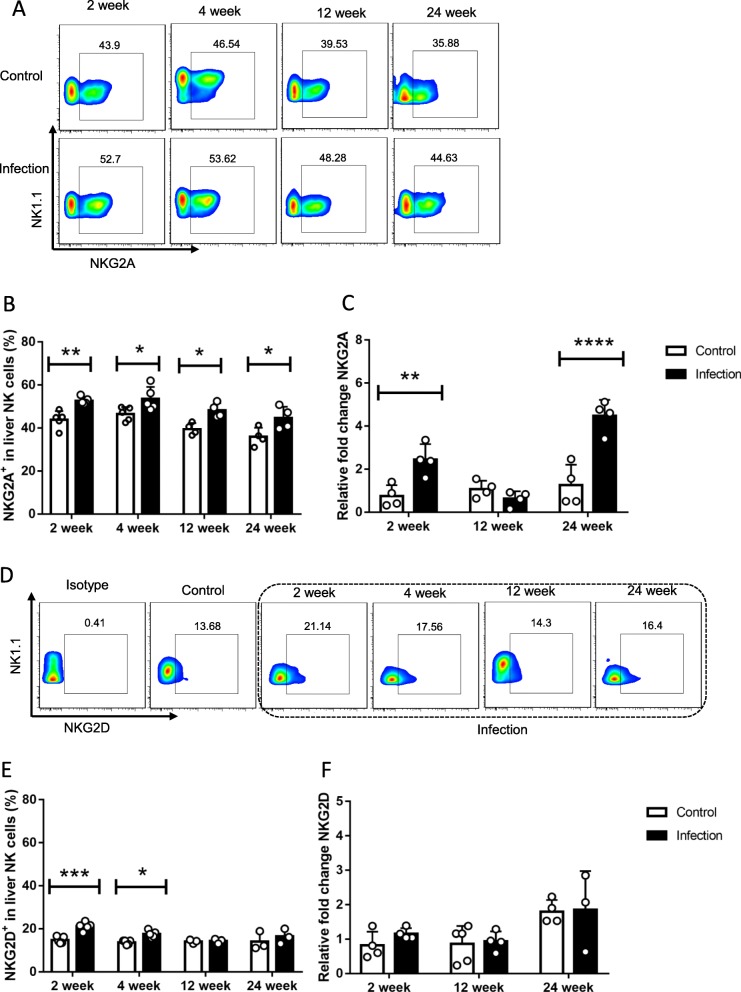
The expression of NKG2A and NKG2D on the hepatic NK cells in *E. multilocularis* infection. **a** Representative FACS plots gated on percentage of NKG2A^+^ in liver NK cells during different time points. **b** The percentage of NKG2A^+^ in liver NK cells after infection. **c** QRT-PCR analysis for mRNA levels of receptor NKG2A in whole liver tissue at various time points after infection and normalized by comparison to the housekeeping gene GAPDH mRNA. **d** Representative FACS plots gated on percentage of NKG2D^+^ in liver NK cells. **e** The percentage of NKG2D^+^ in liver NK cells after infection. **f** QRT-PCR analysis for mRNA levels of receptor NKG2D in whole liver tissue at various time points after infection and normalized by comparison to the housekeeping gene GAPDH mRNA. Data were shown as mean ± standard error (SEM, 3–5 mice per group). *p < 0.05; **p < 0.01; ***p < 0.001, ****p < 0.0001

NK cells’ target recognition, immune response and tolerance are mainly mediated through cell surface receptors, including activating and inhibitory receptors [31]. To investigate whether the receptor alterations affected those changes above, FACS analysis was performed mainly focusing on known surface markers. Relevant data indicated that, expression of inhibitory receptor NKG2A on NK cells was significantly higher at designated time points than control mice (Fig. 5a, b, c), while activating receptor NKG2D was only elevated at early stages (2, 4 weeks) post *E. multilocularis* infection (Fig. 5d, e, f), and similar findings on some other receptors also had no obvious alterations (Additional file 3: Figure S2A, B, C, D). It can be inferred from the scenario that NK cells’ numeric and functional changes might be related to cell surface inhibitory receptor NKG2A molecule dynamics.

### NKG2A^+^ NK cell population in infected mice showed impaired cytokine production

**Fig. 6:**
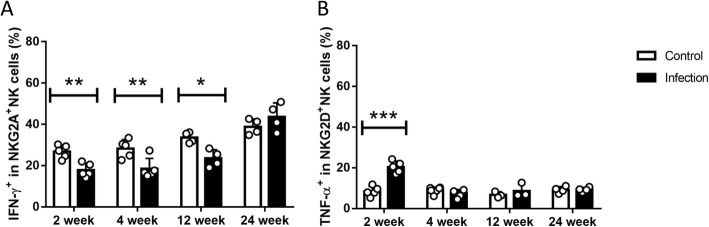
Cytokine production of NKG2A+ NK and NKG2D+ NK cells in E. multilocularis infection. **a** Percentage of IFN-γproduced by liver NKG2A+ NK cells during the different time points after infection. **b** Percentage of TNF-α produced by liver NKG2D+ NK cells during the different time points after infection. Data were shown as mean ± standard error (SEM, 3–5 mice per group), *p values< 0.05, **p < 0.01 and ***p < 0.001

Activated NK cells mediates protection against pathogens, which is characterized through secretion of cytokines, primarily IFN-γ [32]. For the next step, both NKG2A^+^ NK and NKG2D^+^ NK cells were studied for functional analysis to estimate the cytokine production in *E. multilocularis* infection, intracellular cytokines were stained using FACS techniques (Fig. 6). It can be revealed that, NKG2A^+^ NK cells’ IFN-γsecretion was significantly lower than control group, however, which had no obvious change in NKG2D^+^ NK cells, and some other cytokines did the same way (Additional file 4: Figure S3). So far, we were able to demonstrate that NKG2A^+^ NK cell population showed impaired cytokine production in *E. multilocularis* infected mice.

## Discussion

Our results demonstrated the profound decrease of liver NK cells population but sharp increase of NKG2A expression in *E. multilocularis* infected mice. Such alteration might result in low cytotoxic activity through decreased IFN-γ secretion, as well as the disease progression with its potentiality in the immune tolerance milieu in infected liver. To the best of our knowledge, this is the first report on hepatic NK cells and their related molecules in murine model of *E. multilocularis* infection.

NK cells play significant role against intracellular viruses and bacteria, tumors, protozoa, parasites infection [33]. Immune tolerance due to compromised NK cells’ cytotoxic activity has been frequently observed both in experimental and human study with tumor and chronic infection [31, 34]. Since NK cell population play a key role in the process of tumor and chronic infection development, many recent studies support the importance of NK cells during parasitic infection. *Toxoplasma gondii* infection impairs NK cell recognition of target cells and cytokine release, then independently enhances its survival [35]. NK cells can limit malaria infection by producing IFN-γ and killing infected cells before being superseded by the adaptive immune response [22]. Moreover, reduction of NK cell numbers and impaired NK cells response observed in patients with acute cutaneous *leishmaniasis* [36]. NK cells population and its function changed in *Angiostrongylus cantonensis* infected mice, suggesting their involvement in pathogenesis of the infection [24]. Activated NK cells is reported to negatively regulate egg-induced liver fibrosis via producing IFN-γ, and killing activated stellate cells in *Schistosoma japonicum* infection [25]. Like other metacestode infection, AE is featured by chronic granuloses formation and present various immunopathological processes. Available data showed inhibited activation of NK cells after co-culture with *E. multilocularis* vesicular [27]. Human study demonstrated decreased NK cells frequency in peripheral blood in AE patients and concluded the possible involvement of NK cells during AE infection [37]. Herein, our results displayed decrease in NK cell percentage and IFN-γproduction in hepatic tissue of *E. multilocularis* infected mice. It is known that CD49a^+^DX5^−^ and CD49a^−^DX5^+^ are the two major hepatic NK subsets. CD49a^+^DX5^−^ NK cells is liver resident NK cells and possesses memory potential [38]. In current study, lower percentage of CD49a^+^DX5^−^ NK cells were observed in infected liver. And most intriguingly, significant increase of parasite load but decrease of peri-parasitic fibrosis was also observed after the depletion of NK cells.

Commonly, the ingestion of *E. multilocularis* metacestode initiate cross-talk with the host’s immune system and induces recruitment and infiltration of various immune cell types. The fibrotic layer, secondary to the immune response, successfully separates the parasite and limits its growth [39, 40]. In the context of NK cell depletion, the process of cross-talk is not “smooth” and lead the continuous growth of the parasite and failure of limitation by fibrotic tissues. Besides, the over expression of TGF-β [40], and IL-10 [41] after *E. multilocularis* infection might contribute to NK cell inhibition, resulting into NK cell activating receptors’ imbalance, consequently reducing IFN-γ secretion and impairing the NK cell immune surveillance.

NK cell function is tightly regulated by activating and inhibitory molecules [33]. Either the downregulation of activating receptors such as NKG2D, NKp30, NKp46, or upregulation of inhibitory receptors like Tim-3, NKG2A, PD-1 lead to NK cell dysfunction [26]. Upregulation of NKG2A in chronic HBV and HCV infection is purportedly associated with NK cell exhaustion [42, 43]. NKG2A^high^ status contributes to NK cell exhaustion and predicts poor prognosis in liver cancer patients [12]. In addition, newest research demonstrated that anti-NKG2A mouse antibody is an immune checkpoint inhibitor that promotes anti-tumor immunity by unleashing both T and NK cells [44] . In this study, our result showed that NKG2A is upregulated at all designated time points in experimental *E. multilocularis* infection. Such change is related with decreased numeric and functional NK cells at early and middle stages, and it might be responsible for the increased parasite load and extensive infection. However, at the late stage, NK cells’ functional decrease has been reversed, which may be related to server T cell exhaustion led to upregulation of other activated molecules on NK cells in very late stage of infection. Thus, further studies need to work on its explicit mechanism.

NK cells exert its function on pathogens, tumor cells, stressed hepatocytes, and HSCs via the production of cytokines (IFN-γ, TNF-α, IL-10, IL-12, IL-22, etc.) and cytotoxic molecules (granzyme B, Perforin, etc.) both in direct or indirect fashion [26]. The over-expressed NKG2A in hepatitis and HCC patients showed markedly reduction of IFN-γ secretion. Moreover, the NKG2A blockade resulted with significant boosts of IFN-γ production and interdependent of NK and CD8^+^ T cell functions to prevent in hepatitis and HCC patients [44, 45]. Besides, NKG2A downregulation increases the anti-tumor activity of NK cells and infusions in a subset patient with HCC [46]. Human liver-derived CXCR6^+^ NK cells are predominantly educated through NKG2A and show reduced cytokine production [47]. NKG2A downregulation enhanced NK cytotoxicity and accelerates effective treatment responses in patients with chronic myeloid leukemia [48]. Notably, IFN-γ produced by NK cells plays predominant role during anti-viral [19], anti-fibrosis [49], and anti-tumor [50], anti-parasitic process [24]. And, its secretion is inhibited by over expression of TGF-β and IL-10 and therefore related with immune tolerance [51]. High levels of plasma IL-10 is related with over expression of NKG2A and lead to decreased secretion of IFN-gamma and cytotoxicity [12]. In line with abovementioned results, treatment with anti- NKG2A resulted with increased production of NK cells secreted IFN-γ in vivo study [44]. Da-Zhong Shi reported the possible curative effect of IFN-γ both in human and experimental study and indicated the importance of IFN-γ during *E. multilocularis* infection [52]. Further, our results showed the significant reduction of regional levels of NK cell secreted IFN-γ in *E. multilocularis* infection and negative correlation with NKG2A expression.

There are still some inherent limitations of current study should be stated. First, our results were based upon experimental model that might not be the best option but the result currently available. The failure to access commercialized antibody of NKG2A kept us from deeper understanding the possible role of it during *E. multilocularis* infection, however, this would be done in our future work in support to current findings.

## Conclusions

Our study illustrated the significant reduction of liver NK cells population but obvious upregulation of NKG2A expression in *E. multilocularis* infected mice. Such opposite alteration might be related to the impaired production of INF-γ, as well as the disease progression with its potentiality in the immune tolerance milieu in infected liver. Further investigations should be made to identify the more explicit role of NKG2A in liver NK cells exhaustion, which would be very helpful to restoring NK cell related immunity against *E. multilocularis* infection.

## Additional files

Additional file 1: **Table S1.** Fluorescent labeled antibodies for flowcytometry. (DOCX 16 kb)

Additional file 2: **Figure S1.** The decline in hepatic NK cells’ functions in *E. multilocularis* infection. (PPTX 301 kb)

Additional file 3: **Figure S2.** . The expression of activated and inhibitory receptors on the hepatic NK cells in *E. multilocularis* infection. (PPTX 532 kb)

Additional file 4: **Figure S3.** The cytokine production of hepatic NKG2A^+^ NK and NKG2D^+^ NK cells after *E. multilocularis* infection. (PPTX 566 kb)

## Data Availability

The datasets used and/or analyzed during the current study available from the corresponding author on reasonable request.

## Abbreviations

ELRA: Ex vivo liver resection followed by autotransplantation technique
HCC: Hepatic cellular cancer
IFN-γ: Interferon-γ
IL-10: Interleukin-10
NK cells: Natural killer cells
NKG2A: Natural killer group 2, member A
NKG2D: Natural killer group 2, member D
PBMC: Peripheral blood mononuclear cell
PBS: Phosphate buffer saline
TGF-β: Transforming growth factor-β
TNF-α: Tumor necrosis factor—α
α-SMA: α smooth muscle actin
