# Supplementary material for: Echinococcus multilocularis inoculation induces NK cell functional decrease through high expression of NKG2A in C57BL/6 mice
Source: BMC Infect Dis. 2019 Sep 9;19:792. doi: 10.1186/s12879-019-4417-1 (PMC6734356; doi:10.1186/s12879-019-4417-1)
Supplement: Supplementary file 1 — Table S1. Fluorescent labeled antibodies for flowcytometry. (DOCX 16 kb) [file 12879_2019_4417_MOESM1_ESM.docx]

Table S1. Fluorescent labeled antibodies for flow cytometry

| **Antibody Name** | **Fluorescent Substance** | **Clone No.** | **Manufacturer's name** |
| --- | --- | --- | --- |
| NK-1.1 | PE-Cy7 | PK136 | Biolegend |
| CD3 | PerCP-Cy5.5/ FITC/ APC-Cy7 | 17A2 | Biolegend |
| CD69 | APC-Cy7 | H1.2F3 | Biolegend |
| CD27 | Pacific Blue™ | LG.3A10 | Biolegend |
| CD11b | PerCP-Cy5.5 | M1/70 | Biolegend |
| CD49a | APC | HMα1 | Biolegend |
| DX5 | APC-Cy7 | DX5 | Biolegend |
| Ly49D | PE | 4E5 | Biolegend |
| NKG2A | APC | 16A11 | Biolegend |
| NKG2D | PE | CX5 | Biolegend |
| Ly-49G2 | FITC | 4D11 | ebioscience |
| Ly-49H | APC | 3D10 | ebioscience |
| Ly-49I | FITC | YLI-90 | ebioscience |
| granzyme B | FITC | GB11 | Biolegend |
| IFN-γ | PE/ APC | XMG1.2 | Biolegend |
| TNF-α | PerCP-Cy5.5 | MP6/XT22 | Biolegend |
