# Supplementary figures and images for: Echinococcus multilocularis inoculation induces NK cell functional decrease through high expression of NKG2A in C57BL/6 mice
Source: BMC Infect Dis. 2019 Sep 9;19:792. doi: 10.1186/s12879-019-4417-1 (PMC6734356; doi:10.1186/s12879-019-4417-1)

## Slide 1
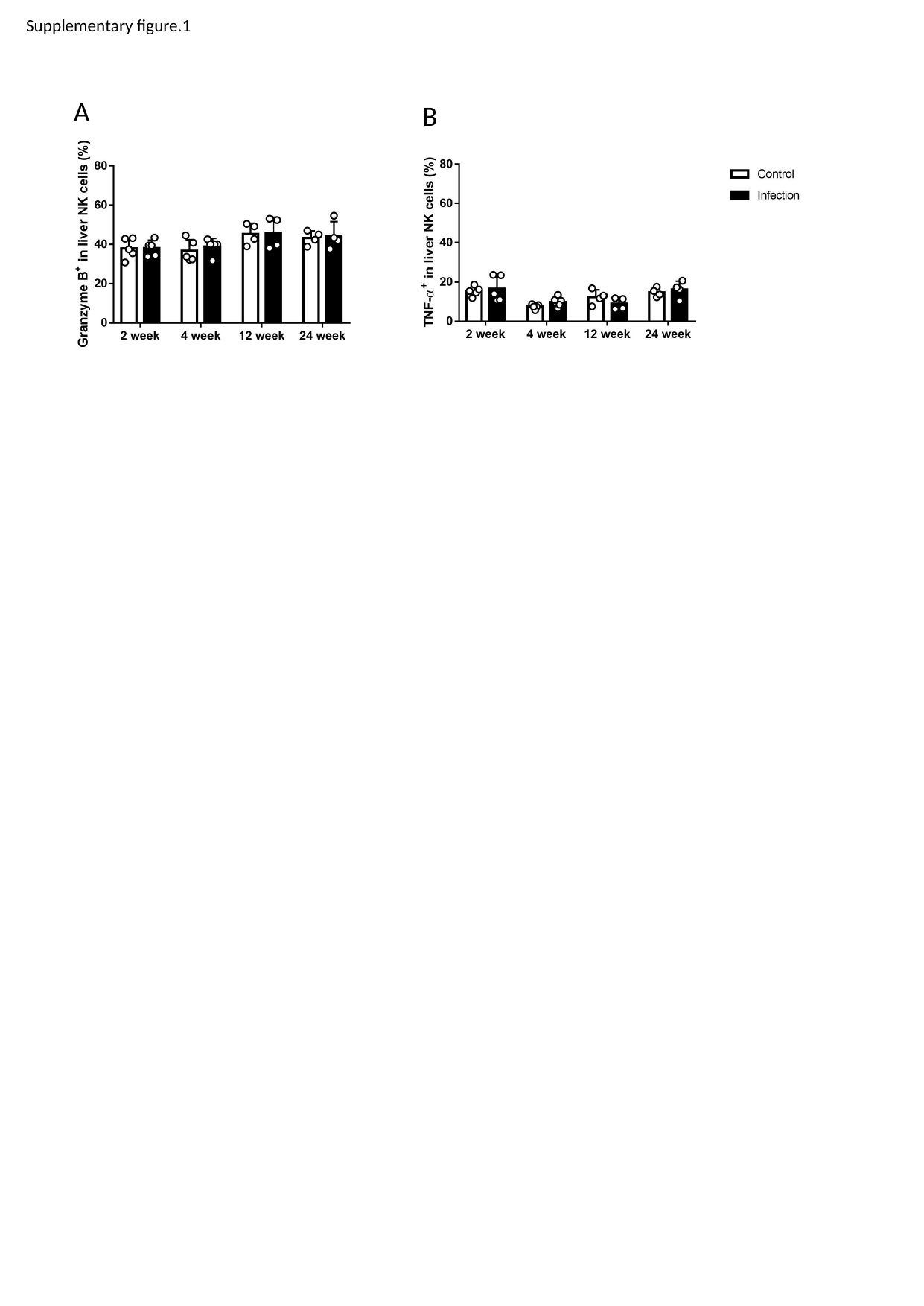

Supplementary figure.1
A
B

Supplement: Supplementary file 2 — Figure S1. The decline in hepatic NK cells’ functions in E. multilocularis infection. (PPTX 301 kb) [file 12879_2019_4417_MOESM2_ESM.pptx]

## Slide 1
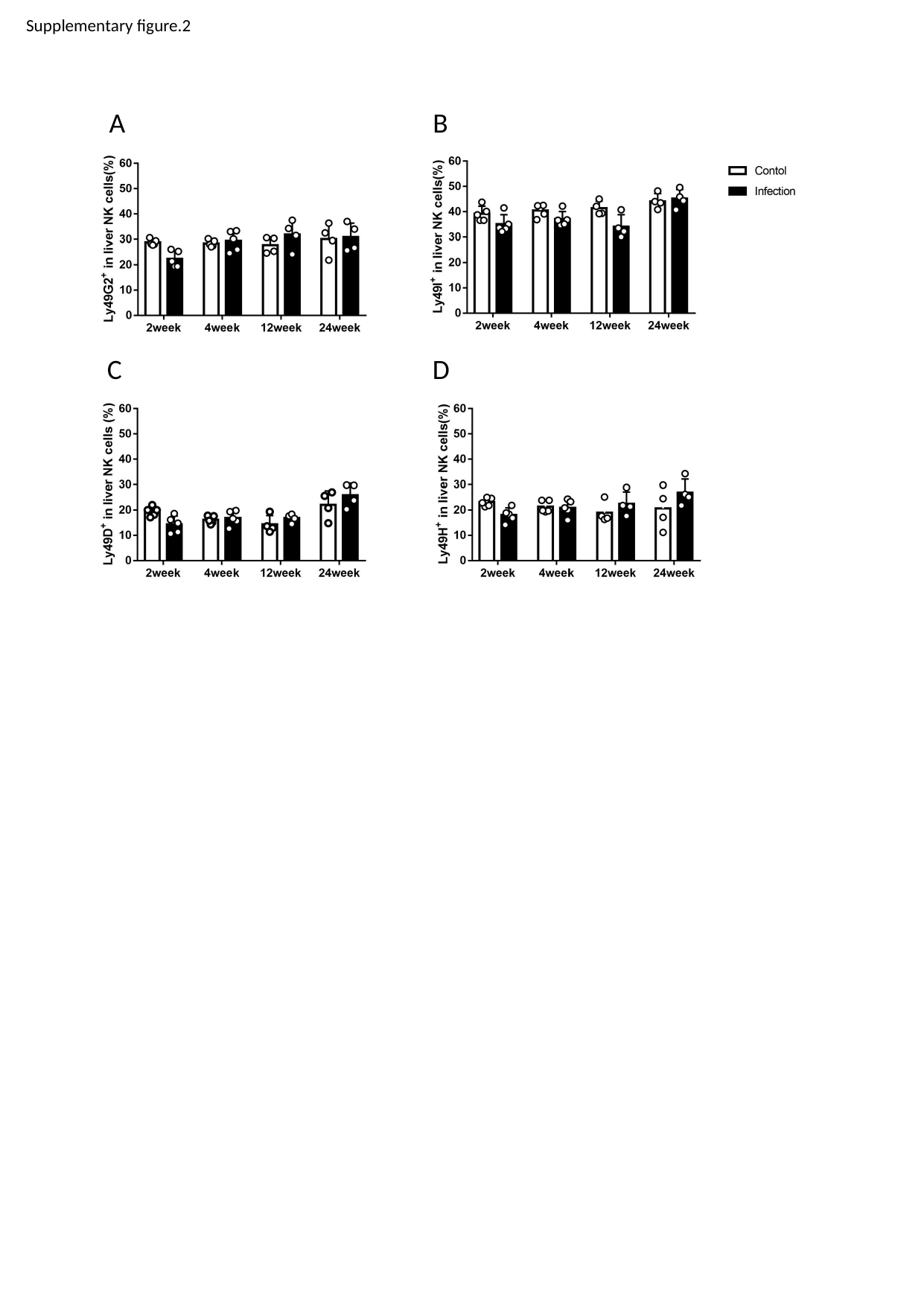

Supplementary figure.2
B
A
D
C

Supplement: Supplementary file 3 — Figure S2. . The expression of activated and inhibitory receptors on the hepatic NK cells in E. multilocularis infection. (PPTX 532 kb) [file 12879_2019_4417_MOESM3_ESM.pptx]

## Slide 1
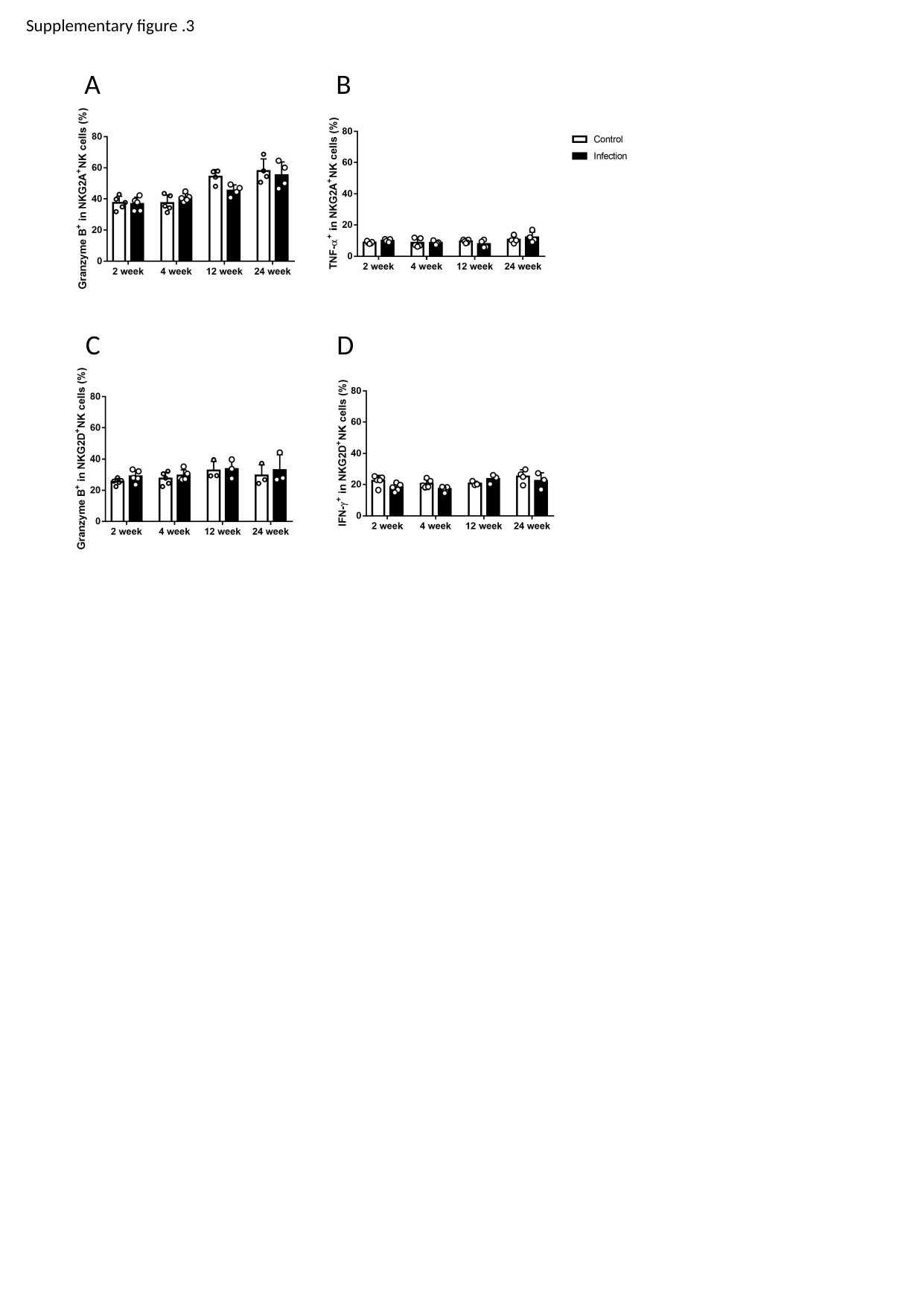

Supplementary figure .3
B
A
D
C

Supplement: Supplementary file 4 — Figure S3. The cytokine production of hepatic NKG2A+ NK and NKG2D+ NK cells after E. multilocularis infection. (PPTX 566 kb) [file 12879_2019_4417_MOESM4_ESM.pptx]
